# Moral distress and burnout in internal medicine residents

**Published:** 2017-02-24

**Authors:** Sharareh Sajjadi, Monica Norena, Hubert Wong, Peter Dodek

**Affiliations:** 1 University of British Columbia, British Columbia, Canada; 2 Center for Health Evaluation and Outcome Sciences, St. Paul’s Hospital and University of British Columbia, British Columbia, Canada; 3 Faculty of Medicine, University of British Columbia, British Columbia, Canada; 4 Division of Critical Care Medicine, St. Paul’s Hospital and University of British Columbia, British Columbia, Canada

## Abstract

**Background:**

Residents frequently encounter situations in their workplace that may induce moral distress or burnout. The objective of this study was to measure overall and rotation-specific moral distress and burnout in medical residents, and the relationship between demographics and moral distress and burnout.

**Methods:**

The revised Moral Distress Scale and the Maslach Burnout Inventory (Human Service version) were administered to Internal Medicine residents in the 2013–2014 academic year at the University of British Columbia.

**Results:**

Of the 88 residents, 45 completed the surveys. Participants (mean age 30+/−3; 46% male) reported a median moral distress score (interquartile range) of 77 (50–96). Twenty-six percent of residents had considered quitting because of moral distress, 21% had a high level of burnout, and only 5% had a low level of burnout. Moral distress scores were highest during Intensive Care Unit (ICU) and Clinical Teaching Unit (CTU) rotations, and lowest during elective rotations (p<0.0001). Women reported higher emotional exhaustion. Moral distress was associated with depersonalization (p=0.01), and both moral distress and burnout were associated with intention to leave the job.

**Conclusion:**

Internal Medicine residents report moral distress that is greatest during ICU and CTU rotations, and is associated with burnout and intention to leave the job.

## Introduction

The Royal College of Physicians and Surgeons of Canada considers well-being of physicians as an aspect of Professionalism, one of the seven CanMEDS roles for physicians in Canada. Therefore, well-being of residents is part of the Royal College’s accreditation standards for residency training programs.^1^ Reports of distress and burnout start from the earliest stages of training. For example, Canadian medical students report higher level of stress than their peers.^2^ In addition, depression and anger are reported more frequently as medical trainees progress through their internship year.^3^ And depression during internship is associated with burnout during residency.^4^

Burnout is defined as a “state of vital exhaustion” by the World Health Organization International Classification of Diseases (ICD-10).^5^ Maslach described burnout as a combination of emotional exhaustion from overwhelming work demands, depersonalization, and reduced perceived personal accomplishment.^6^,^7^ Emotional exhaustion is caused by overwhelming work demands, depersonalization leads to an impersonal response toward patients or other health care providers, and a perceived lack of personal accomplishment can be caused by fatigue.^7^ West showed that Internal Medicine (IM) residents report high levels of emotional exhaustion (46%) and depersonalization (20%).^8^

Residency is a stressful period of medical training.^9^ Due to long work hours, reported lack of support and control, work-home interference, and sleep deprivation,^10^ residents in various specialty training programs report depression, stress, and anxiety.^10^–^12^ For example, burnout occurs in up to 76% of IM residents.^13^ In a study related to ours, burnout and impostorism were reported in 12% and 44%, respectively, of University of Western Ontario IM residents.^14^ In a related study of family practice residents, Michels et al. found a higher prevalence of burnout in men, Caucasians, and third year residents.^15^

These disturbances affect not only the well-being of the residents, but also have an adverse effect on patient care.^13^,^16^ Fatigue and exhaustion in residents is associated with self-reported medical errors, suboptimal medical care, poor quality of life, and burnout.^10^,^17^–^19^ In addition to burnout, distress has been associated with decreased quality of life.^13^

Residents and medical students also face situations in their training where they may experience moral distress. Moral distress is “the painful psychological disequilibrium that results from recognizing the ethically appropriate action, yet not taking it, because of such obstacles as lack of time, supervisory reluctance, an inhibiting medical power structure, institutional policy, or legal considerations.”^20^,^21^ The traditional hierarchy of medical teams may foster this problem.^22^ Moral distress was first described in nurses by Jameton in 1984.^23^ He described the psychological disequilibrium and painful feelings caused by conscious inaction because of institutional burdens such as lack of support, time, and legal limits. Subsequently, Corley used a one-item visual analogue scale and showed that 80% of critical care nurses in her setting report high level of distress.^24^ One of Corley’s colleagues, Hamric, developed and validated the revised Moral Distress Scale.^25^ Using this scale, moral distress has been identified in nurses, physicians, and other health professionals who work in Intensive Care Units in British Columbia.^26^

Although burnout in practicing physicians^26^–^28^ and residents^9^,^10^,^13^,^16^ has been the subject of multiple studies, moral distress has been studied mostly in nurses^20^,^21^,^29^–^31^ and there are only a few studies that have included physicians.^30^,^32^ Most recent studies of moral distress have been done in the intensive care unit (ICU).^26^,^32^–^34^ Moral distress in nurses has been linked to burnout and attrition.^29^,^35^,^36^ We are not aware of any studies of moral distress in residents including those enrolled in IM training programs in North America.

Therefore, the objective of this study was to measure moral distress and burnout in IM residents using validated instruments, the revised Moral Distress Scale and the Maslach Burnout Tool.^7^,^25^,^37^ We examined the associations between moral distress and specific types of rotations, the relationship between scores on these two instruments and characteristics of the residents, and the relationship between moral distress and burnout in this population. Our hypotheses were that moral distress is higher during ICU and Clinical Teaching Unit (CTU; IM ward) rotations than during elective rotations, and that moral distress is associated with burnout. The basis of the former hypothesis is that, compared to other rotations, we believed that residents would experience more situations during ICU and CTU rotations that are potential causes of moral distress.

## Methods

### Potential study participants

At the end of the 2013–2014 academic year (June, 2014), there were 159 residents, from the first to the fourth post-graduate year (PGY1 to PGY4) level, enrolled in the University of British Columbia (UBC) IM training program. In the UBC IM curriculum, there are 20 weeks of CTU, 4 weeks of ICU, and 4 weeks of Cardiac Care Unit (CCU) during the first post graduate year; 12 weeks of CTU and 8 weeks of CCU during the second year; and 16 weeks of CTU and 8 weeks of ICU during the third year. The remainder of the academic year consists of elective rotations in different subspecialties.^38^

During the last three academic half-day sessions in June 2014, the end of academic year 2013–2014, we distributed self-administered surveys to all 88 residents who voluntarily attended these sessions. Residents who did not attend these academic half-day sessions were excluded from this cross-sectional study because our Research Ethics Board prohibited us from sending the survey by electronic mail or other means directly to these residents. We also could not provide any formal briefing for residents because of Research Ethics Board limitations.

### Respondents

Forty-five of 88 IM residents (51%) who attended the academic half-day sessions completed the surveys. Out of 45 returned surveys, 43 could be analyzed; two surveys were excluded because respondents answered fewer than 10 items on the surveys and one survey did not disclose the age of the responder. Table 1 shows the demographics of respondents. Analysis was done with non-missing values ranging from 38 to 42 observations depending on the variable analyzed.

### Data collection

Participation in this study was voluntary and all data were anonymous. Surveys were handed out at the three main hospital sites where academic half-days were held, and residents returned the surveys into collection boxes at academic half-day sessions or to the postgraduate office or research representatives at their convenience. If a resident chose to return the survey to the postgraduate office, they would leave the survey in a box containing both completed and uncompleted surveys, keeping their anonymity protected. Furthermore, personnel in the postgraduate office were not involved in the handling or analysis of the surveys.

In addition to completing the items on the surveys, residents were asked to state their sex, age, ethnic background (East Asian, South Asian, Caucasian, Other) and postgraduate year (1, 2, 3, 4).

### Study Measures

To measure moral distress, we used the revised Moral Distress Scale.^25^,^37^ This survey contains 21 items which are descriptions of situations. For each item, the respondent uses a Likert-scale to report frequency of the situation and the associated level of disturbance (0–4 corresponds to low-high for each). For each item, the two Likert scores are multiplied and the sum of the 21 products is the moral distress score. A higher value means more frequent and/or more intense moral distress. For this survey, we asked residents to consider situations during only the most recent academic year. This time frame allowed us to examine the effect of level of training on moral distress.

In addition to the standard items on the survey, the residents were also asked to rate the frequency and level of disturbance related to moral distress during their ICU, CTU, CCU, and elective rotations during the most recent academic year. We also asked participants whether they had ever considered quitting a clinical position, or were currently considering leaving their position because of their moral distress with the way patient care was handled at their institutions.

To measure burnout, we used the Maslach Burnout Inventory (MBI), which has been validated for use in residents.^37^ We used the Human Service version of MBI, known as MBI-HSS. This instrument has 22 items, which are the elements of the three domains of burnout: emotional exhaustion (EE), depersonalization (DP), and personal accomplishment (PA). For each item, residents rated the frequency of experiencing various feelings or emotions on a 7-point Likert scale from “never” to “daily”. Higher scores for EE and DP, and lower scores for PA point to burnout.^7^,^12^,^39^ Based on standards from the MBI developer, an EE score of 27 or over is categorized as high, 17–26 is moderate, and 0–16 is low. A DP score of 13 and over is categorized as high, 7–12 is moderate, and 0–6 is low. And a PA score of 39 or over is categorized as high, 32–38 is moderate, and 0–31 is low.^7^

### Statistical analysis

For normally distributed data, we used parametric tests (unpaired t-test, analysis of variance, Pearson correlation) and for non-normally distributed data, we used the Wilcoxon signed rank sum test. We used unpaired t-tests to compare scores for moral distress and burnout between the two sexes, and between those who had considered quitting due to moral distress and those who had not. We used analysis of variance to compare moral distress and burnout scores among the various levels of postgraduate training and the four ethnic groups. We used the Wilcoxon signed rank sum test to compare levels of moral distress in ICU, CTU, CCU, and elective rotations. We calculated the Pearson correlation coefficient for the relationship between the moral distress score and each element of the Maslach Burnout Inventory. We also calculated this coefficient for the relationship between age and each of the scores (moral distress, EE, DP, PA) in which each respondent was the unit of analysis.

Approval to conduct this study was provided by the UBC-Providence Health Care Research Ethics Board.

## Results

### Moral distress

The overall median (interquartile range (IQR)) moral distress score was 77 (50, 96); the possible range was 0–336. There were no relationships between moral distress and age, sex, ethnicity, or postgraduate year (p=0.32, p=0.48, p=0.20 and p=0.81 respectively). However, there were differences among the moral distress scores associated with each of the four types of rotations. Both ICU and CTU rotations had the highest median scores of 4 (IQR 1, 9 for ICU and IQR 2, 9 for CTU; the maximum possible score for each was 16), followed by CCU with a median score of 2 (IQR 1, 9) and elective rotations which had a median score of 1 (IQR 0, 2). Although there was no statistically significant difference between scores associated with ICU and CTU rotations (p=0.23), moral distress scores associated with ICU and CTU rotations were higher than those associated with CCU rotations (p=0.025 and p=0.012, respectively). Moral distress scores associated with ICU and CTU rotations were also greater than those associated with elective rotations (p<0.0001 for both).

Eleven residents (26%) had previously considered quitting their clinical position because of moral distress related to the way patient care was handled at their institution, but only two respondents (5%) were at the time of the survey considering leaving their position.

### Burnout

The mean (SD) score for emotional exhaustion (EE) was high at 27.6 (9.5). The mean score for depersonalization (DP) was 11.9 (5.8), which is in the moderate category, and the mean score for personal accomplishment (PA) was 34.3 (7.7), which is high. Only 5 residents (12%) scored low on EE, whereas 21 (49%) scored high and 17 (39%) scored moderate. Similarly, only 9 residents (21%) reported low levels of DP, but 20 (46%) reported high and 14 (33%) reported moderate levels of DP. On the other hand, 14 residents (32.5%) reported high levels of PA, 15 (35%) reported moderate levels, and 14 (32.5%) reported low levels of PA.

High level of burnout is defined by high scores on the EE and DP subscales and low scores on the PA subscale;^17^ nine residents (21%) met criteria for a high level of burnout. Only 2 residents (5%) reported a low level of burnout, indicated by a low level of EE and DP and a high level of PA; the remaining 74% of the respondents scored between these extremes.

Although there were no statistically significant relationships between moral distress or burnout, and age or year of residency, women reported higher EE (30) than men (24; p=0.03). In addition, South Asian respondents reported higher PA than other ethnic groups (p=0.05).

### Relationship between Moral distress or Burnout Scores and Intention to quit the job

Higher scores for moral distress, and for the emotional exhaustion and depersonalization components of burnout were all related to intention to quit the job due to the distress caused by the way patient care was handled at the resident’s institution (Table 2).

In addition, there was a positive correlation between the moral distress score and the depersonalization component of burnout (r=0.39, p=0.01). The associations between moral distress and emotional exhaustion (r=0.29, p=0.06) or personal achievement (r=0.07, p=0.7) were not statistically significant.

## Discussion

We found that medical residents experience moral distress and that this distress is especially prominent during ICU and CTU rotations. Furthermore, more than a quarter of our sample had considered quitting their job due to moral distress and a similar proportion experienced substantial levels of burnout.

Moral distress was correlated with burnout, but was not related to age, sex, or postgraduate year of the residents. Emotional exhaustion, one of the components of burnout, was greater in women than in men. These results raise concerns about the well-being of internal medicine residents in general, but especially in certain rotations, and for women.

Medical trainees expect stress and long work hours as a part of their training program but there is increasing evidence that physicians experience emotional suffering and pathologic distress that may affect their professionalism and care of their patients.^40^ The current study adds to this body of knowledge by demonstrating moral distress and burnout in medical residents.

Compared to CCU and elective rotations, we found that moral distress was significantly higher during ICU and CTU rotations. The reason for this observation may be that ICU and CTU rotations have high turnover of the sickest patients and where residents have longer work hours and may experience lack of support. This interpretation is consistent with previous studies of nurses in which sicker patients, lack of time, and low staffing have been linked to moral distress.^23^,^41^

These findings call for further studies to identify the causes of moral distress in residents to identify potential solutions. Factors that contribute to moral distress in nurses include insufficient numbers of staff, inadequately trained staff, and organizational policies, such as ineffective pain control orders, that make it hard for nurses to meet the needs of families and patients.^41^ The most recent Canadian study on moral distress showed that nurses are suffering from higher moral distress than staff physicians in the ICU setting.^26^ Medical residents face challenges that are similar to those of nurses - perceived lack of support and challenge of the hierarchy within the medical team (attending physicians leading the care) but there are currently no data to illuminate the causes of moral distress in this population.^10^

Jacobson and Redman described the link between unresolved ethical problems and reduced job satisfaction, burnout, and high staff turnover.^42^,^43^ An example of this link is that five to 25 percent of nurses who participated in Corley’s studies left their position as a result of moral distress.^20^,^44^ Similarly, in our study, 26% of respondents were considering leaving their position as a result of their moral distress. Millette pointed to powerlessness as a major factor that influences nurses who change their position.^45^ In our study, moral distress (which can be due to powerlessness), emotional exhaustion, and depersonalization were all associated with the tendency to quit the job. Similarities between our results and those in studies of nurses are expected because both residents and nurses report feeling a lack of control and stress despite their important and irreplaceable services and responsibilities. Both professions also experience long work hours and lack of support.^10^,^41^

In our study, 79% of resident respondents reported some degree of burnout and 21% reported a high degree of burnout as indicated by abnormal levels of all three domains of the MBI-HSS. This is consistent with previous studies from the United States which reported that up to 76% of residents experience some degree of burnout.^10^,^13^ Our result is also compatible with a study of Family Practice residents at UBC in 2012, which showed a 70% burnout rate.^46^ Although our cross-sectional analysis precludes any inferences about causality, the relationship that we observed between moral distress and depersonalization suggests that moral distress may cause this component of burnout.

In our study, women reported a higher degree of emotional exhaustion, and South Asians reported a higher degree of personal achievement. These findings have not been reported in previous studies of burnout in residents; in addition, none of the previous studies were done in Canadian settings.^10^

The consequences of moral distress and burnout are serious. For example, both can affect patient care through more self-reported medical errors, inappropriate care of patients, increased length of stay, and escape-avoidance strategies in the affected health care provider that are directed towards patients.^17^,^46^–^49^ These findings indicate that the mental health of health care providers is an important priority for the health care system.

Although this is the first Canadian study on moral distress and burnout in residents which points to the association between these phenomena and intention to quit the job, our study is limited by its cross-sectional design, small sample size, and likely inadequate statistical power to detect some of the differences that we were examining. In addition, we relied on the memory of the residents about their experience over the preceding year to assess their level of distress and burnout, and there are no standards against which to validate these measures of moral distress. In addition, our study is limited to one institution and one specialty; the results may not be generalizable to other settings or specialty trainees. Finally, we cannot rule out selection bias because we distributed the surveys only to the residents who voluntarily attended the academic half days; this approach was due to the constraints of our research ethics board. Nevertheless, we had a response rate of 51%. It is possible that moral distress and burnout may be different in the residents who did not attend academic half-day sessions and were not able to volunteer to participate in our study.

We are not aware of any formal curricula in the Canadian IM residency programs and affiliated hospitals to enable residents with strategies to recognize their feelings and address their emotional needs. Residents contribute significantly to the work done in university-affiliated hospitals, often being the front-line providers in caring for the sickest patients. This responsibility may predispose the residents to feelings of frustration, fatigue, anxiety, and based on our study, moral distress and burnout. These findings require attention to recognize these phenomena and develop appropriate interventions to address moral distress and burnout in residents. Further longitudinal and multi-centre studies would also help to identify the causes of distress and burnout in residents.

## Figures and Tables

**Table 1:** Respondents’ characteristics and survey scores

	All Respondents	First Year	Second Year	Third Year	Fourth Year
**N**	40	14	12	13	1

**Age Mean (SD)**	30 (3)	28 (2)	30 (2)	31 (3)	32 (−)

**Male/Total (% Male)**	18/40 (45)	11/14 (79)	3/12 (25)	4/13 (31)	0/1 (0)

**Ethnicity**					
**Caucasian**	21	8	5	8	0
**East Asian**	9	5	3	1	0
**South Asian**	2	0	0	1	1
**Others**	6	0	4	2	0

**Moral Distress Median (IQR)**	77 (50, 96)	78 (67, 96)	80 (50, 90)	88 (64, 120)	49 (49, 49)

**Emotional Exhaustion**	26.5 (20, 36)	29 (18, 38)	25 (17, 34.5)	27 (22, 31)	32 (32, 32)

**Depersonalization**	12 (7, 16)	13.5 (8, 16)	7.5 (4.5, 14)	14 (8, 16)	15 (15, 15)

**Personal Accomplishment**	35.5 (30, 40)	35.5 (28, 40)	36 (31, 38.5)	34 (31, 38)	40 (40, 40)

**Table 2:** Relationship between Propensity to Quit the Job, and Moral Distress and Burnout Scores

Response to question about quitting position due to moral distress	Moral Distress	Emotional Exhaustion	Depersonalization	Personal Accomplishment
**No, I’ve never considered quitting or left a position**	73.5 (33.9)*	25.3 (8.9)	10.6 (5.4)	34.6 (8.1)
**Yes, I considered quitting but did not leave**	99.4 (33.1)*	34.4 (8.5)	15.5 (5.8)	32.8 (6.4)
***p*-value from T-test**	0.03	0.005	0.01	0.50

* mean (standard deviation)
